# High-Throughput Phenotyping and Random Regression Models Reveal Temporal Genetic Control of Soybean Biomass Production

**DOI:** 10.3389/fpls.2021.715983

**Published:** 2021-09-03

**Authors:** Fabiana Freitas Moreira, Hinayah Rojas de Oliveira, Miguel Angel Lopez, Bilal Jamal Abughali, Guilherme Gomes, Keith Aric Cherkauer, Luiz Fernando Brito, Katy Martin Rainey

**Affiliations:** ^1^Department of Agronomy, Purdue University, West Lafayette, IN, United States; ^2^Department of Animal Sciences, Purdue University, West Lafayette, IN, United States; ^3^Department of Agricultural and Biological Engineering, Purdue University, West Lafayette, IN, United States; ^4^Department of Statistics, Purdue University, West Lafayette, IN, United States

**Keywords:** digital agriculture, *Glycine max*, longitudinal traits, phenomics, plant breeding, time series, quantitative genetics

## Abstract

Understanding temporal accumulation of soybean above-ground biomass (AGB) has the potential to contribute to yield gains and the development of stress-resilient cultivars. Our main objectives were to develop a high-throughput phenotyping method to predict soybean AGB over time and to reveal its temporal quantitative genomic properties. A subset of the SoyNAM population (*n* = 383) was grown in multi-environment trials and destructive AGB measurements were collected along with multispectral and RGB imaging from 27 to 83 days after planting (DAP). We used machine-learning methods for phenotypic prediction of AGB, genomic prediction of breeding values, and genome-wide association studies (GWAS) based on random regression models (RRM). RRM enable the study of changes in genetic variability over time and further allow selection of individuals when aiming to alter the general response shapes over time. AGB phenotypic predictions were high (*R*^2^ = 0.92–0.94). Narrow-sense heritabilities estimated over time ranged from low to moderate (from 0.02 at 44 DAP to 0.28 at 33 DAP). AGB from adjacent DAP had highest genetic correlations compared to those DAP further apart. We observed high accuracies and low biases of prediction indicating that genomic breeding values for AGB can be predicted over specific time intervals. Genomic regions associated with AGB varied with time, and no genetic markers were significant in all time points evaluated. Thus, RRM seem a powerful tool for modeling the temporal genetic architecture of soybean AGB and can provide useful information for crop improvement. This study provides a basis for future studies to combine phenotyping and genomic analyses to understand the genetic architecture of complex longitudinal traits in plants.

## Introduction

Soybean [*Glycine max* (L.) Merr.] is one of the most economically important crops worldwide, being the primary source of plant-based protein, and the second largest source of vegetable oil (USDA, 2018). Advances in plant breeding and agronomic methods have substantially improved soybean yield over time (Anderson et al., 2019). Yield potential in any environment or cropping system can be expressed as a function of biomass produced, and the partitioning of biomass to the seeds, or harvest index (Monteith, 1972, 1977). Assessments of historical soybean germplasm have shown that increases in soybean grain yield over the last several decades are associated with increases in biomass production (Cregan and Yaklich, 1986; Frederick et al., 1991; Kumudini et al., 2001; De Bruin and Pedersen, 2009; Koester et al., 2014; Balboa et al., 2018). For instance, Koester et al. (2014) measured above-ground biomass (AGB) every 2 weeks in cultivars released between 1923 and 2007 and observed that biomass production per unit of absorbed light increased with the release year. Additionally, information on temporal biomass production provides insights into crop development and responses to multiple abiotic and biotic stressors (Bajgain et al., 2015; Jumrani and Bhatia, 2018). Increased temperatures and water stress have imposed vegetative and reproductive stage reduced AGB significantly and resulted in 28% and 74% reduction in soybean yield, respectively (Jumrani and Bhatia, 2018). Hence, understanding the genetic factors controlling the temporal dynamics of biomass accumulation may contribute to future soybean yield gains and the development of stress-resilient cultivars.

Measuring crop AGB across developmental stages is laborious, involving cutting, drying, and weighing plants from a target area, and is subject to errors and limitations resulting from (1) unrepresentative samples; (2) destructive sampling, which limits the number of samples that can be collected from a plot, and prevents longitudinal tracking of the same target area; and (3) extensive manual handling, which may lead to sample loss, and can be restrictive in large experiments (Jimenez-Berni et al., 2018). High-throughput phenotyping platforms (HTPP) offer alternatives to ground-based AGB sampling, enabling collection of non-destructive data throughout the growing season in large experiments under actual field conditions (van Eeuwijk et al., 2018; Zhao et al., 2019). In some crops, such as wheat, barley, rice, and dry beans, AGB accumulation has been recognized as a potential target to increase yield gain, and the success of image-based AGB phenotyping has been demonstrated (Serrano et al., 2000; Babar et al., 2006; Tilly et al., 2014; Cheng et al., 2017; Neumann et al., 2017; Yue et al., 2017; Sankaran et al., 2018). In soybean, Maimaitijiang et al. (2019) used red, green and blue (RGB) imagery-derived metrics to predict AGB in production fields; however, there are no studies on the use of HTPP to estimate soybean AGB in experimental plots with different genotypes used for plant breeding.

High-throughput phenotyping (HTP) allows time-series measurements that monitor the development of a crop through its life stages, and how it responds to the environment (Moreira et al., 2020). These measurements represent the crop in different “ages” or stages of development, with the mean and variance between measurements usually changing over time, characterizing the trait as longitudinal (Falconer and Mackay, 1996; Yang et al., 2006; Oliveira et al., 2019a). In animals, it has been shown that the phenotypic or additive polygenic effects of longitudinal traits are not constant during expression of longitudinal traits (Szyda et al., 2014; Brito et al., 2018; Oliveira et al., 2019a), so that breeders need an amenable statistical framework for genetic and genomic analysis that accounts for time-dependent genetic contributions to the phenotypes of longitudinal traits.

Different approaches can be utilized for genomic evaluation of longitudinal traits (Moreira et al., 2020). A simple repeatability (SR) model treats the individual measurements recorded over time as repeated records of the same trait (Meyer and Hill, 1997). This model assumes that the variances of different measurements are equal and the genetic correlations between all measurements are equal to one, which is an unrealistic assumption for most crop studies (Falconer and Mackay, 1996; Meyer and Hill, 1997; Littell et al., 1998). An alternative method that overcomes these restrictions is a multiple-trait model (MTM), which treats individual measurements over time as different traits. However, high-dimensional longitudinal data can lead to high correlations between consecutive measurements and over-parameterized models with high computational demands, restricting the application of MTM (Foster et al., 2006; Speidel, 2011). Random regression models (RRM) provide a robust framework for estimating breeding values and identifying alleles with time-specific effects for longitudinal traits (Oliveira et al., 2019a; Moreira et al., 2020) In summary, RRM use a given covariance function to describe the trajectory of the trait as a function of time (or environmental gradient), with no assumptions for constant variances and correlations (Kirkpatrick et al., 1990; Meyer and Hill, 1997; Schaeffer, 2016). RRM have some key advantages compared to other models, such as (1) greater computational efficiency, (2) prediction of breeding values for any time point within the range of data collection, and (3) more accurate breeding values (Oliveira et al., 2019a). RRM were originally proposed for use in livestock breeding programs and have been successfully used for genetic evaluation of longitudinal traits (Jamrozik and Schaeffer, 1997; Schaeffer, 2004; van Pelt et al., 2015; Englishby et al., 2016; Oliveira et al., 2019a), but have only recently been implemented in crops (Sun et al., 2017; Campbell et al., 2018, 2019). Thus, we hypothesized that RRM can be efficiently used to model temporal measurements of complex polygenic traits in crops.

In this context, this study aimed to: (1) develop an HTTP methodology to estimate soybean AGB throughout the growing season; (2) reveal the genetic architecture and estimate time-dependent effects of single-nucleotide polymorphisms (SNPs) associated with this longitudinal trait using RRM; and (3) investigate the feasibility of implementing genomic selection for longitudinal traits in soybean using RRM.

## Materials and Methods

### Plant Materials, Field Experiments, and Genotypic Data

We used a set of 383 recombinant inbred lines (RILs) representing 32 families from the Nested Association Mapping (SoyNAM) population (~12 RILs per family; Diers et al., 2018). The lines comprising the set were selected using breeding values for full maturity (R8; Fehr and Caviness, 1977) and grain yield, calculated from experiments performed in Indiana and Illinois from 2011 to 2014, in order to have a maturity-controlled panel (Xavier et al., 2016; Lopez et al., 2019). More details about the RIL panel selection and the full list of traits’ collection and distribution are described in Lopez et al. (2019).

The RILs were grown under a randomized complete block design with two replications at the Purdue University Agronomy Center for Research and Education (ACRE), West Lafayette, IN, United States (40°28'20.5”N 86°59'32.3”W) and Romney, IN, United States (40°14'59.1'N 86°52'49.4'W). Planting occurred on May 31, 2017 and May 22, 2018 at ACRE, and May 17, 2018 at Romney. Soil fertility information and environmental conditions summarized by days after planting (DAP) for this experiment are described in Lopez et al. (2019). The combination of year and location where the experiment was grown was considered as an environment, resulting in three environments in this study (2017_ACRE, 2018_ACRE, and 2018_Romney). Experimental units consisted of a six-row plot (3.35 m with 0.76 m) with a targeted seeding rate of 35 seeds m^−2^. A total of 66 and 16 RILs were discarded in 2017 and 2018, respectively, because of poor emergence. In addition to the two full replications, we randomly selected 62 RILs in 2017 and 108 RILs in 2018 (the same 62 RILs in 2017 plus 46 others) to grow in a trail of eight-row plots (0.76 m × 3.35 m). This trail was defined as the biomass sampling panel and it was used as sampling plots for destructive AGB measurements throughout the growing season.

**Figure 1:**
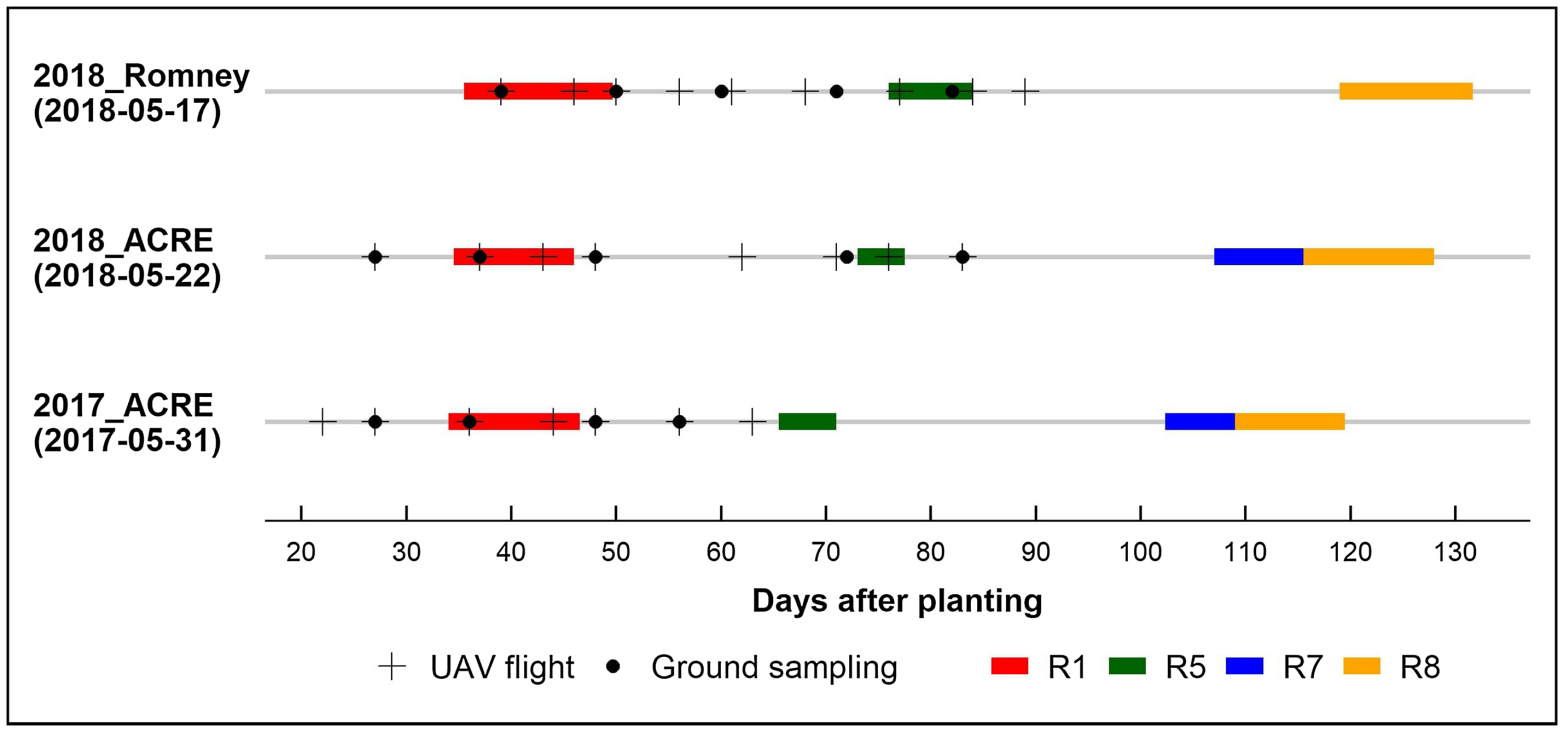
Data collection timeline by environment 2017_ACRE, 2018_ACRE, and 2018_Romney. Planting date in parentheses below environment. UAV: unmanned aerial vehicle. Phenological stages (Fehr and Caviness, 1977): R1, beginning bloom; R5, beginning seed; R7, beginning maturity; and R8, full maturity.

In the biomass sampling panel, AGB was collected approximately every 10 days during the growing season between 27 to 83 DAP, from a linear section of 0.56 m in a row with borders. In 2017, we randomly picked plots to measure AGB in replication one for every sampling date, while in 2018, three full AGB sampling (~38, 58, and 84 DAP) were performed for both locations in the two full replications. The fresh AGB was dried at 80°C using a dry-air system until achieving constant weight. Finally, we obtained the dry AGB weight and rescaled it to g/m^2^. Figure 1 shows the data collection timeline for each environment and the respective phenological stage periods.

The SoyNAM founder parents were genotyped by Song et al. (2013) using the SoySNP50K BeadChip resulting in 42,509 segregating SNP markers that were imputed into the SoyNAM RILS using the Williams 82 reference genome (Wm82.a2.v1) bp positions by Diers et al. (2018). For genotypic quality control, we excluded SNPs with minor allele frequencylower than0.05 and call rate lower than 0.90, resulting in 40,110 SNPs for the genome-wide analyses.

### High-Throughput Phenotyping

RGB and multispectral imagery were collected with fixed-wing SenseFly eBee unmanned aerial vehicle (UAV). RGB imagery was collected using a S.O.D.A. camera (SenseFly Parrot Group, Switzerland). Multispectral imagery was collected with a 1.2 MP Parrot Sequoia camera (MicaSense Inc., Seattle, United States), which captures four discrete spectral bands: green (wavelength = 550 nm, bandwidth = 40 nm), red (660 nm, 40 nm), red-edge (735 nm, 10 nm), and near-infrared (790 nm, 40 nm). Flights were performed close to solar noon at an altitude of approximately 120 m with both RGB and multispectral cameras. The forward and side overlap for flights were set to at least 85 and 70%, respectively. Ground control points were installed at the corners of the trials and their GPS coordinates were recorded using the TOPCON RTK (Topcon, Tokyo, Japan).

To process the multispectral imagery from this experiment, two pipelines were built in MATLAB: Crop Image Extraction version 2 (CIE 2.0) and Vegetation Indices Derivation version 1 (VID 1.0; Lyu et al., 2019). The multispectral images were stitched using Pix4Dmapper (Pix4D SA, 2018) to produce a full ortho-mosaic of the experimental area. Individual plots were extracted from the ortho-mosaic using the CIE 2.0. Segmentation was performed to highlight the canopy of the vegetation using the Otsu’s method (Otsu, 1979). Radiometric calibration was done for every sampling date to remove atmospheric effects and potentially correct for any sensor sensitivity issues (Iqbal et al., 2018). During flight operations, we laid out four reflectance panels reflecting at a specific and consistent percentage of light (12, 22, 36, and 48% reflectance). A handheld spectrometer ASD FieldSpec^®^ 4 (ASD, Boulder, CO, United States) was used to measure the true reflectance of the panels while the multispectral images were collected. We used the reflectance values from the panels, along with radiance values of the panels, extracted from the generated ortho-mosaics, to correct the radiance values for the plots using the empirical line method (Smith and Milton, 1999), which is crucial in producing reflectance data over the plots. The reflectance from the calibrated images was used to calculate vegetation indices (VI) using the VID 1.0 pipeline. Vegetation indices are typically used to estimate crop biomass, and for this study, we selected 14 VIs (Supplementary Table 1) previously reported in the literature to correlate with crop biomass (Babar et al., 2006; Bendig et al., 2015; Wang et al., 2016; Yue et al., 2017; Sankaran et al., 2018).

From the RGB imagery, we calculated canopy coverage (CC) using the software Progeny^®^ (Progeny Drone Inc., West Lafayette, IN, United States) and the multilayer mosaic approach as described by Hearst (2019). The list of the imagery features used in this study is in Supplementary Table 1. All imagery features were calculated in intact and bordered plot rows not used for destructive biomass sampling.

### Predicting Above-Ground Biomass

To predict the AGB for all DAP, including days when ground truth data were not available, we considered a linear model using the imagery features as the predictor variables within each environment across all observed DAP. We observed that the distribution of the residuals was highly asymmetric, suggesting that a linear model was not suitable to fit the data (Thoni et al., 1990). To correct the asymmetry, we considered a Box-Cox transformation on the AGB, which led to the log-transformed values (data not shown, Box and Cox, 1964). The prediction of AGB was carried out using two different machine-learning methods: Least Absolute Shrinkage and Selection Operator (LASSO) Regression (Tibshirani, 1996) and Partial Least Squares Regression (PLSR; Wold et al., 2001). Both methods have been commonly used in building predictive models with HTP data (Montes et al., 2011; Bratsch et al., 2017; Wang et al., 2017; Vasseur et al., 2018; Fu et al., 2019).

Regularization methods, such as LASSO, can reduce model complexity using a “penalty” parameter that minimizes the sum of squared error. As such, LASSO performs both regularization and variable selection, by shrinking variable coefficients to zero, and eliminating variables from the model when their coefficients reach zero. The PLSR is an extension of the multiple linear regression and principal component analysis that can also effectively handle the issue of multicollinearity among predictor variables (Wold et al., 2001). Essentially, PLSR performs simultaneous decomposition of the predictor and response variables into latent variables and then identifies key components that explain covariance between them (Abdi, 2010). For the PLSR, 10 principal components were selected so that the root mean squared error (RMSE) from cross-validation was minimized.

The performance of the predictive models was evaluated using a 10-fold cross-validation strategy, in which the dataset was randomly divided into a training set (90% of the plots) and validation set (10% of the plots). The predictive accuracy of the model was measured by the coefficient of determination (*R*^2^), which is equal to the fraction of AGB variance explained by the model, and by the RMSE, which measures the average error magnitude. Pearson’s correlation coefficient (*r*) was also considered to quantify the linear correlation between the observations and their estimates, being an indication of model prediction ability. Both models were implemented in the R software (R Core Team, 2019), using the package *caret* (Kuhn, 2008).

### Random Regression Models

RRM were used to model AGB across 27 to 83 DAPs. Seven different models were tested: third-, fourth-, and fifth-order Legendre orthogonal polynomials (Kirkpatrick et al., 1990) and linear and quadratic B-splines (de Boor, 1980; Meyer, 2005) with one (at 55 DAP) or two knots (at 44 and 66 DAPs). In RRM, Legendre orthogonal polynomials and B-splines (segmented polynomials joined by knots) are used to describe the covariance structure of the data as a function of time (de Boor, 1980; Kirkpatrick et al., 1990; Meyer, 2005).

The general RRM can be described as:

$$y_{ijk}=Env_{k}+\overset{m}{\underset{m=1}{\sum }}b_{m}\emptyset_{m}\left(t_{ij}\right)+\overset{m}{\underset{m=1}{\sum }}a_{im}\emptyset_{m}\left(t_{ij}\right)+e_{ijk},$$

where *y_ijk_* is the predicted AGB of the *i^th^* RIL on DAP *j* within environment and replication combination *k*; *Env_k_* is the fixed effect of environment and replication combination; *b_m_* is the *m* fixed regression coefficient for modeling the average curve of the population; *a_im_* is the *m* random regression coefficient that describes the additive genetic effects for the *i^th^* RIL; *t_ij_* is the time of data collection (DAP *j*) for the *i^th^* line; $\emptyset_{m}\left(t_{ij}\right)$ is a regression function according to DAP *j* (using Legendre or B-spline polynomials); and *e_ijk_* is the random residual effect. The number of regression coefficients *m* varies according to the functions used for random regressions. For the Legendre orthogonal polynomials, $\emptyset_{m}\left(t_{ij}\right)$ is the *m^th^* Legendre orthogonal polynomial coefficient for DAP *j* (standardized for the −1 to 1 interval) from RIL *i*. In the case of B-splines, $\emptyset_{m}\left(t_{ij}\right)$ is the *m^th^* interval given the previously mentioned knots associated with DAP from RIL *i*. According to Meyer (2005), the basis function of degree *p*=0 has values of unity for all points in a given interval (t) and zero otherwise. For the *m^th^* interval given by knots $T_{m}$ and $T_{m+1}$, with $T_{m}\leq t<T_{m+1}$, $\emptyset_{m}\left(t_{ij}\right)=\left\{\begin{array}{c} 1,if\;T_{m}\leq t<T_{m+1}\\ 0,otherwise\; \end{array}\right.$. Basis function for p>0 can be represented by $\begin{array}{l} \emptyset_{m,p}\left(t_{ij}\right)=\left(\frac{t-T_{m}}{T_{m+p}-T_{m}}\;\right)\;\emptyset_{m,p-1}\left(t_{ij}\right)\\ +\left(\frac{T_{m+p+1}-t}{T_{m+p+1}-T_{m+1}}\;\right)\;\emptyset_{m+1,p-1}\left(t_{ij}\right) \end{array}$. The individual segments were either linear or quadratic, with degree *p*=1 or 2, respectively. The joined knots allow the function to become continuous.

The models’ assumptions are as:

$$\mathrm{var}\left[\begin{array}{c} a\\ e \end{array}\right]=\left[\begin{array}{cc} G⊗G_{0} & 0\\ 0 & I⊗R \end{array}\right],$$

where **G**_**0**_ is the (co)variance matrix of the genomic random regression coefficients, **G** is a genomic relationship matrix, **I** is an identity matrix, **R** represents a matrix containing residual variances, and ⊗ is the Kronecker product between matrices. The **G** matrix was calculated using the method presented by VanRaden (2008). The residual variances were allowed to be either homogeneous or heterogeneous. We defined a different residual variance for each of the 18 DAP with AGB phenotypic data and grouped the remaining days based on their proximity to those DAP. The 18 heterogeneous residual variances classes are as follow: 27–33, 34–36, 37, 38–41, 42–43, 44–45, 46, 47–49, 50–53, 54–58, 59–61, 62, 63–65, 66–71, 72–74, 75–76, 77–80, 81–82, and 83.

The AIREMLF90 and BLUPF90 software from the BLUPF90 family (Misztal et al., 2002) were used to estimate the variance components and the solutions of the mixed model equations, respectively. The BLUPF90 family programs perform by default the single-step GBLUP (Misztal et al., 2009; Aguilar et al., 2010; Christensen and Lund, 2010); however, as all RILs were genotyped, the program was adapted to perform the traditional GBLUP (VanRaden, 2008), by using a dummy pedigree file. Akaike’s information criterion (AIC; Akaike, 1974) was used to compare the models’ performance, in which models with lower AIC values were preferred.

### Genetic Parameters

The genetic (co)variance matrix (**Σ**) for all DAP within the interval of AGB collection was obtained as (Oliveira et al., 2019a):

$$\Sigma =TGT^{\prime },$$

where **T** is a matrix of covariates associated with the function assumed for RIL *i* and ***G*** is the genetic (co)variance matrix for the coefficients. The narrow-sense heritability, defined as the proportion of phenotypic variance due to additive genetic variation, for each DAP ($h_{j}^{2}$) was obtained as:

$$h_{j}^{2}=\frac{{\hat{\sigma }}_{a_{j}}^{2}}{{\hat{\sigma }}_{a_{j}}^{2}+{\hat{\sigma }}_{e}^{2}}$$

where ${\hat{\sigma }}_{a_{j}}^{2}$ is the additive genetic variance for DAP j and ${\hat{\sigma }}_{e}^{2}$ is the residual variance, which depends on the residual variance classes previously mentioned (when using the heterogeneity of residual variance). The genetic correlation between different DAP ($r_{j,j^{\prime }}$) was obtained as:

$$r_{j,j\prime =}\frac{{\hat{\sigma }}_{a_{j,j\prime }}}{\surd \left({\hat{\sigma }}_{a_{j}}^{2}+{\hat{\sigma }}_{a_{j\prime }}^{2}\right)},$$

where ${\hat{\sigma }}_{a_{j,\;j}}^{2}$is the genetic covariance between the DAP j and j′, and ${\hat{\sigma }}_{a_{j}}^{2}$ and ${\hat{\sigma }}_{a_{j\prime }}^{2}$ are the additive genetic variances for DAP *j* and j′, respectively. The vector of genomic estimated breeding values (${\hat{GEBV}}_{i}$) for all DAP of RIL i was obtained as (Oliveira et al., 2019a):

$$\hat{GEBV_{i}}=T{\hat{g}}_{i},$$

where ${\hat{g}}_{i}$ is the vector of predicted genomic values for the coefficients, for each RILi, and **T** is a matrix of covariates associated with the assumed function.

### Genomic Prediction of Breeding Values

The performance of the genomic prediction of breeding values for AGB was investigated using a 5-fold cross-validation (CV) scheme. Briefly, all RILs were randomly separated into five equal-sized groups, where one group was retained as validation, and four groups were used as training. This procedure was repeated five times, with a unique group used exactly once as the validation set. Variance components and SNP marker effects were estimated based on the training set and used to predict GEBV in the validation set (reduced data). The prediction accuracy was measured using the Pearson’s correlation coefficient (r) estimated between the GEBV predicted using the full data (i.e., data including all training and validation RIL) and the reduced data, only for the validation RIL. To evaluate the genomic prediction bias, regression coefficients (*b_1_*) were estimated using linear regression of the GEBV estimated based on the full dataset on the GEBV estimated based on the reduced dataset from each CV fold ($GEBV_{full}=b_{0}+b_{1}*GEBV_{reduced}$). Finally, prediction bias (*b_1_*) was calculated as the average of CV folds for each DAP.

### Genome-Wide Association Study

For the GWAS, SNP effects were derived from GEBVs for each additive random regression coefficient using the POSTGSF90 software (Aguilar et al., 2014). The prediction of SNP effects (${\hat{u}}_{m}$ **)** for the $m^{th}$ random regression coefficient was calculated as (Wang et al., 2012):

$${\hat{u}}_{m}=DZ^{\prime }{\left(ZDZ\right)}^{-1}{\hat{GEBV}}_{m}$$

where **D** is a diagonal matrix of weights accounting for variances of SNPs markers (assumed as an identity matrix in this study), **Z** is a matrix relating genotypes of each locus, and ${\hat{GEBV}}_{m}$ is the vector of GEBV for the $m^{th}$ random regression coefficient. Finally, the SNP effects for all DAP were obtained as (Oliveira et al., 2019c):

$${\hat{SNP}}_{s}=T{\hat{u}}_{s},$$

where ${\hat{SNP}}_{s}$ is the vector that contains the SNP effects estimated for each DAP of the $s^{th}$ SNP, ${\hat{u}}_{s}$ is the vector of SNP solutions for all random regression coefficients related to the $s^{th}$ SNP, and **T** is a matrix of covariates associated with the assumed function.

The SNPs were selected to be further investigated based on the magnitude of their effects, as suggested by Oliveira et al. (2019c). In this context, the top 10 SNPs that showed the highest magnitude of SNP effect in each DAP were selected as relevant SNPs. The exploration of candidate genes was carried out in the range of ± 25 kb from the location of the selected SNP. Potential candidate genes and their associated functional annotation were determined using the genomic position and gene models based on Glyma.Wm82.a2.v1 genome in the soybean database SoyBase (Soybase, 2020).

## Results

### Predicting Above-Ground Biomass

**Figure 2:**
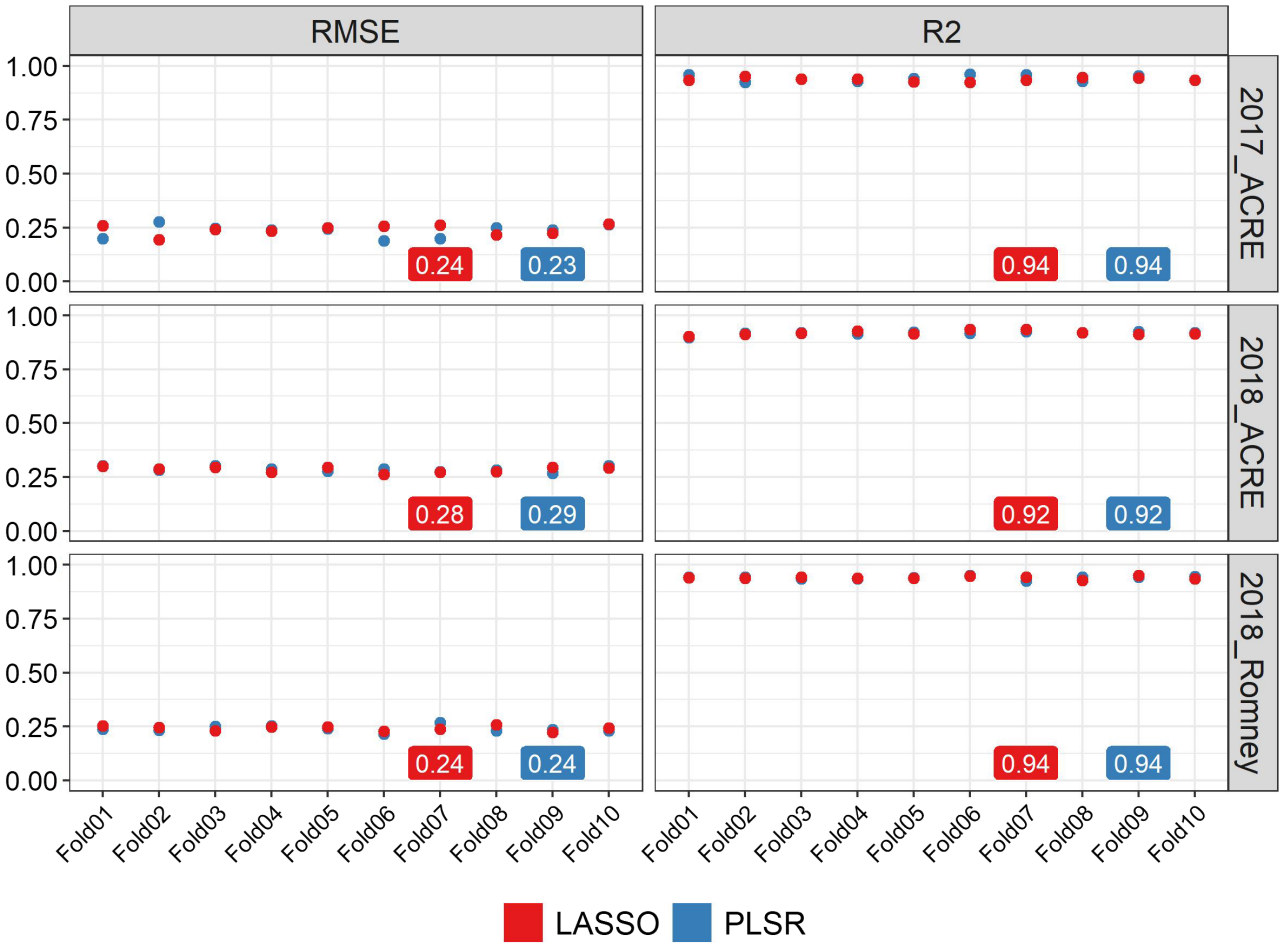
Performance of above-ground biomass prediction for each environment. Predictions were performed using the least absolute shrinkage and selection operator (LASSO) regression, and the partial least squares regression methods. The performance of predictions was evaluated using the root mean squared error (RMSE) and coefficient of determination (R2), using a 10-fold cross-validation set. The y-axis represents the values for RMSE and R^2^ and x-axis indicates each cross-validation fold.

We used two methods to quantify the ability of image-based features to statistically predict the AGB in soybean: LASSO regression and PLSR. Both methods were evaluated using a 10-fold CV strategy and we obtained high prediction performance for AGB estimation with both methods. Figure 2 shows the statistical distributions of *R*^2^ and RMSE values for each CV fold, in each environment. In general, similar performance was observed for both methods in all environments. It was found that LASSO and PLSR had the same R^2^ averages for 2017_ACRE (0.94), 2018_ACRE (0.92), and 2018_Romney (0.94). However, the PLSR presented a smaller RMSE average for 2017_ACRE (0.23 vs. 0.24 for PLSR and LASSO, respectively), and LASSO presented a smaller RMSE average for 2018_ACRE (0.28 and 0.29 for LASSO and PLSR, respectively). Both models presented the same RMSE average for 2018_ACRE (0.24).

The correlation between AGB predicted from UAV-based imagery and observed from ground samples was high (*r* ≥ 0.91) in all environments for both methods, implying that the methods captured the relationship among image-based features and AGB (Supplementary Figure 1). Based on these findings, and because it makes a simpler and more direct connection between the response and predictor variables, the LASSO method was chosen to predict AGB for all plots of the two full replications on all flight dates in this study. Supplementary Figure 2 shows the relative importance of each predictor variable for the LASSO method, which indicates that the model utilized information from different predictor variables for each environment. In addition, we performed a CV leaving one environment out to assess the models’ ability to predict AGB for a new environment. In this scenario, the performance of both methods declined greatly (Supplementary Figure 3). The phenotypic distribution of the predicted AGB across environments and within each environment, by DAP, is presented in Supplementary Figure 4 and Supplementary Figure 5, respectively.

### Genetic Parameters

**Figure 3:**
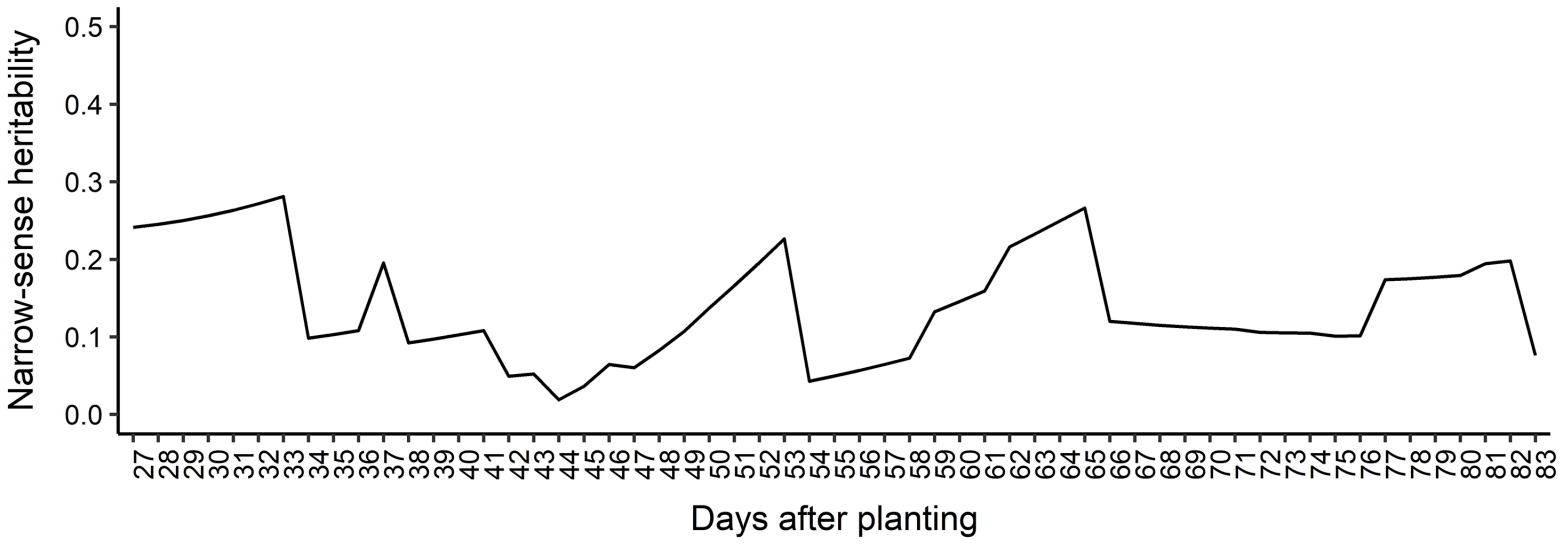
Narrow-sense heritability estimated for each day after planting.

**Figure 4:**
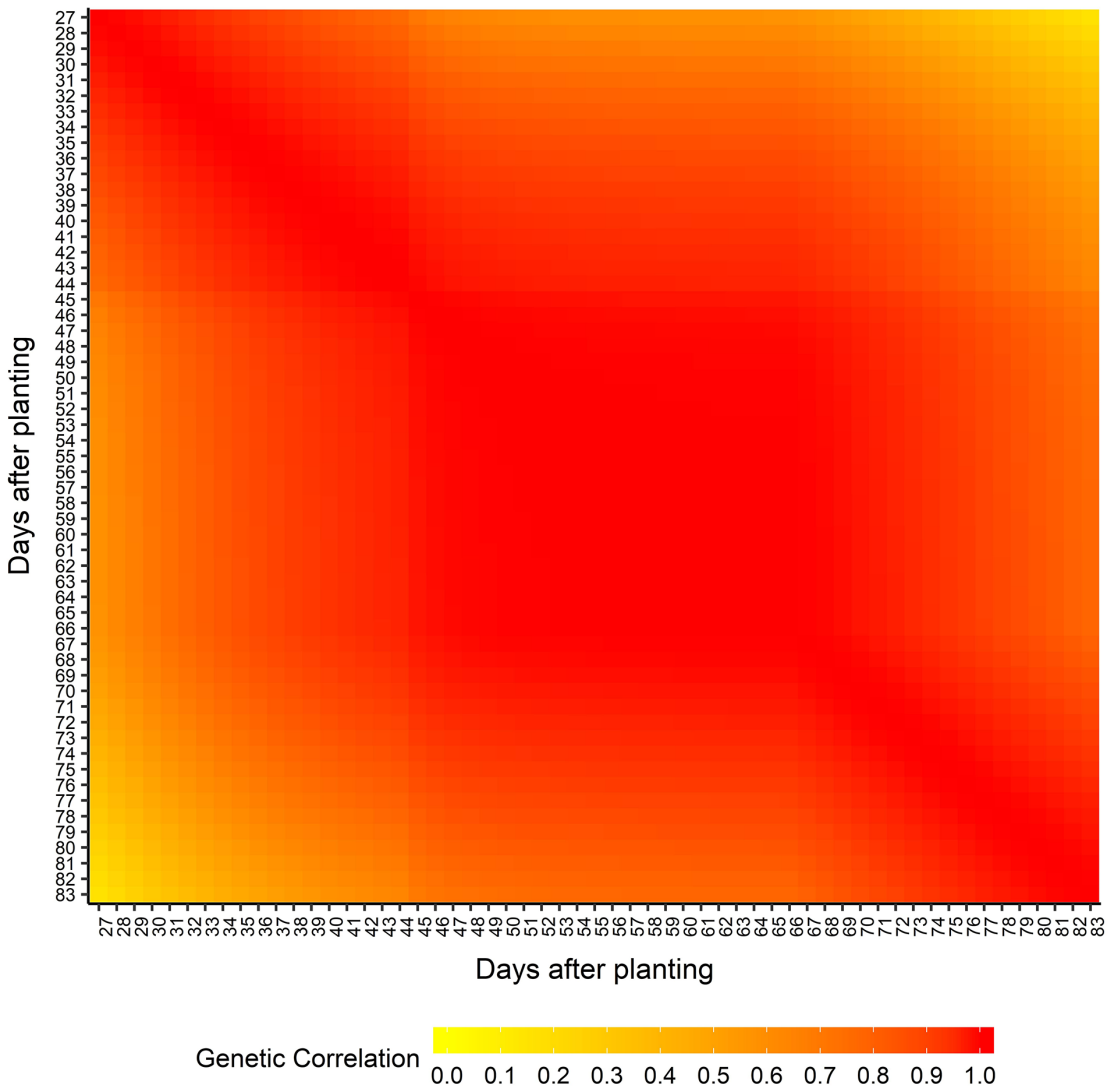
Estimated genetic correlation of above-ground biomass between days after planting.

Supplementary Table 2 shows the AIC values calculated for all seven RRM using both homogeneous and heterogeneous residual variance. The best model was using linear B-spline with 2 knots and heterogeneous residual variance and it was selected for subsequent genome-wide analyses. The genetic architecture of predicted AGB was assessed by estimating the narrow-sense heritabilities (*h*^2^) across the 57 days (from 27 to 83 DAP; Figure 3) from the RRM. Narrow-sense heritability estimates for AGB were low to moderate and varied over time (ranging from 0.02 at 44 DAP to 0.28 at 33 DAP). The genetic correlation between AGB on different DAP was also estimated, and it is showed in Figure 4. Adjacent DAP showed the highest genetic correlations, while those further apart exhibited lower correlations. For instance, the lowest genetic correlation between 27 and 83 DAP was 0.16 and the highest genetic correlation between 48 to 50 DAP was 1.00.

### Genomic Prediction of Breeding Values

**Figure 5:**
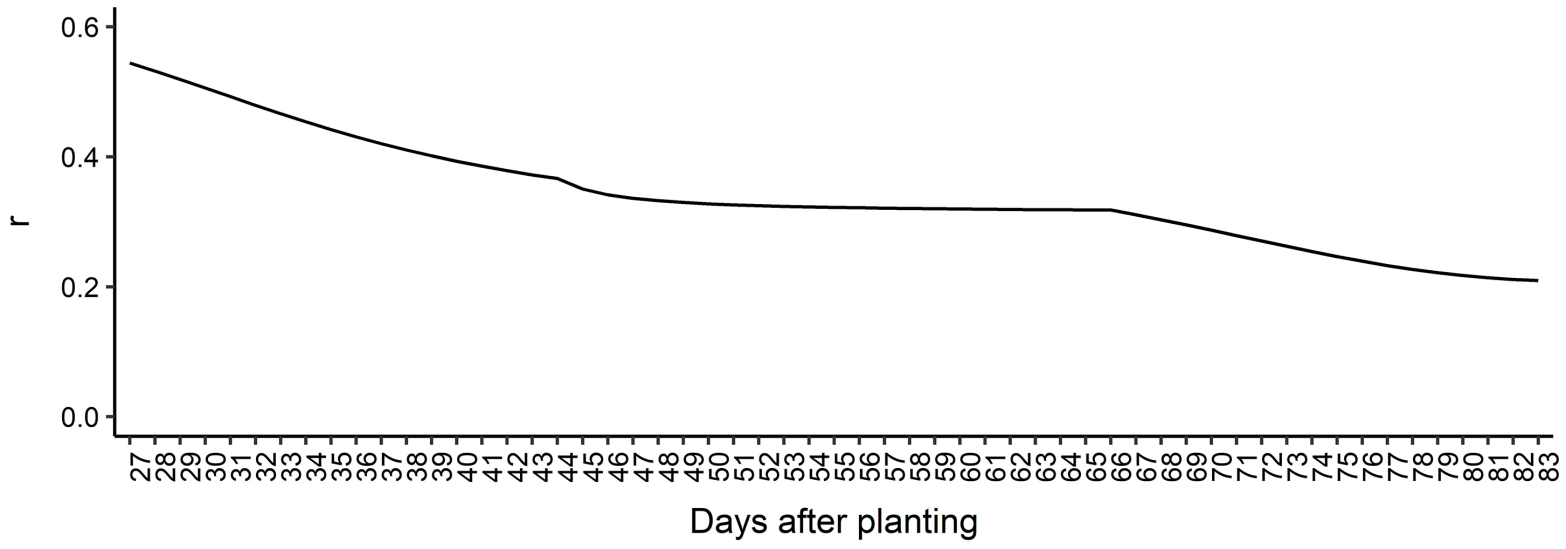
Genomic prediction accuracy based on Pearson’s correlation coefficient (*r*) for each day after planting.

The genomic prediction accuracy for AGB over time is presented in Figure 5. Overall, the prediction accuracies were high considering the heritabilities estimated across all DAP, ranging from 0.21 at 83 DAP to 0.55 at 27 DAP. We observed a decreasing trend in prediction accuracy over time, indicating that it is more difficult to predict AGB for latter DAPs compared to early DAPs. From 27 DAP to 44 DAP, the prediction accuracy steadily decreased, reaching a slight plateau between 44 to 66 DAP, and decreased again until the end of the surveyed time. These findings suggest that longitudinal phenotypes can be accurately predicted using RRM. Regression coefficients’ patterns were used to access the bias of GEBV over DAP (Supplementary Figure 6). Overall, regression coefficients closer to 1.0 were found in earlier DAP. The most biased estimates with regression coefficients deviating from 1.0 were observed toward the end of the surveyed time.

### Genome-Wide Association Study

**Figure 6:**
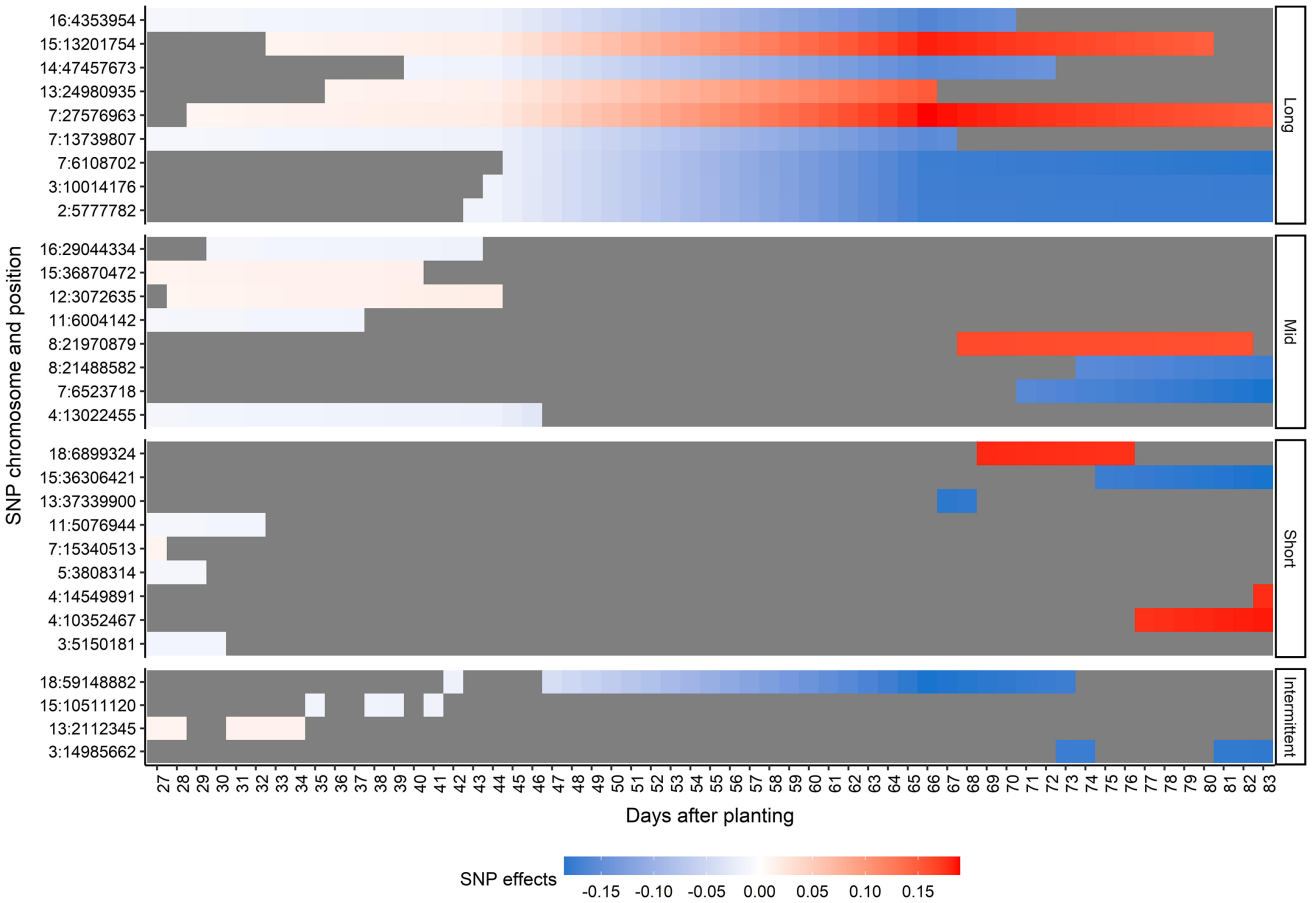
Effects for the selected single-nucleotide polymorphisms (SNPs) across days after planting, in each duration category. Duration categories were defined as long-duration (SNPs present for more than 30 consecutive days), mid-duration (SNPs present for more than 10 consecutive days but less than 30), short-duration (SNPs present for less than 10 consecutive days), and intermittent (SNPs at different non-consecutive intervals). Each y-axis point corresponds to one SNP represented by the chromosome number and position in the soybean Williams 82 reference genome (Wm82.a2.v1; Diers et al., 2018). The blue scale represents negative effects and the red scale represents positive effects. The gray color indicates a zero effect.

Thirty unique SNPs were selected as the most relevant SNPs for AGB. Figure 6 shows the chromosome number, position, period of occurrence, and the SNP effects for selected SNPs. None of the SNPs selected were significant across all time points. In general, the magnitude of effects over time increased for most of the selected SNPs. According to the duration of the SNP effect across all 57 predicted days, the selected SNPs were classified as long-duration (they were considered as important SNPs for more than 30 consecutive days), mid-duration (they were considered as important SNPs for more than 10 consecutive days but less than 30), short-duration (they were considered as important SNPs for less than 10 consecutive days), and intermittent (they were considered as important SNPs on different non-consecutive intervals; Figure 6). These SNP classes were nearly evenly distributed as long- (9 SNPs), mid-(8 SNPs), and short-duration (9 SNPs). The intermittent category had the lowest number of relevant SNPs (4 SNPs). The majority of mid-duration SNPs was detected toward the beginning of the DAP. Interestingly, the SNPs classified in the short- and mid-duration categories were found either toward the beginning or end of the studied time period.

A comprehensive list of positional candidate genes related to the selected SNPs can be found in Supplementary Table 3. As expected, due to the high number of SNPs selected, the number of candidate genes identified was also high. No positional candidate genes within ± 25 kb were found for five selected SNPs: 3:14985662, 4:10352467, 4:14549891, 7:27576963, and 15:36870472. Among the selected SNPs, eight fell within potential candidate genes in the soybean genome (Table 1).

**Table 1:** Selected single-nucleotide polymorphisms (SNPs) associated with above-ground biomass mapped inside potential candidate genes in the soybean genome.

Duration Category	SNP	Chr.	Pos. (bp)	Selected candidate genes	Annotation Description
Long	2:5777782	2	5,777,782	*Glyma.02 g064500*	Rhomboid protein-related
				*Glyma.02 g064600*	Agenet domain-containing protein
Short	3:5150181	3	5,150,181	*Glyma.03 g040800*	Regulator of chromosome condensation (RCC1) family with FYVE zinc finger domain
Long	7:6108702	7	6,108,702	*Glyma.07 g067900*	Disease resistance protein (TIR-NBS-LRR class), putative
Mid	7:6523718	7	6,523,718	*Glyma.07 g071800*	cytidine/deoxycytidylate deaminase family protein
Short	7:15340513	7	15,340,513	*Glyma.07 g128300*	–
Long	13:24980935	13	24,980,935	*Glyma.13 g137200*	ROP interactive partner 3
Short	15:36306421	15	36,306,421	*Glyma.15 g217500*	CTP synthase family protein
Long	16:4353954	16	4,353,954	*Glyma.16 g046000*	DEAD/DEAH box helicase, putative

Chr, Chromosome; Pos (bp), position in base pair; – annotation not available.

## Discussion

### High-Throughput Phenotyping of Soybean Above-Ground Biomass

Besides being an important yield component, plant biomass is a foundation for unraveling several complex processes of plant growth, development, and environmental response (De Bruin and Pedersen, 2009; Koester et al., 2014; Balboa et al., 2018; Jumrani and Bhatia, 2018). The capacity to non-destructively estimate soybean AGB enables capturing these data in a temporal fashion leading to insights about AGB dynamics. Previously, satellite-derived vegetation indices were used separately to predict soybean AGB with high predictive abilities (Kross et al., 2015; Richetti et al., 2019). However, both studies are from production fields with no significant genetic variation. Recently, Maimaitijiang et al. (2019) used UAV-based RGB imagery-derived spectral, structural, and volumetric information to predict AGB in production fields with three cultivars, but the study did not represent the genetic diversity or small plot formats typical of breeding programs. To our best knowledge, this is the first study estimating soybean AGB of experimental plots and diverse genotypes, demonstrating the feasibility to measure and use this trait in plant breeding programs.

Many different techniques and HTPP have been used to estimate AGB in different crops (Bendig et al., 2015; Wang et al., 2016; Zhang et al., 2017; Jimenez-Berni et al., 2018; Maimaitijiang et al., 2019). Using information from multiple sensors is a common practice to predict AGB because it improves trait estimation by combining the advantages of the spectral, spatial, and structural metrics derived from different sensors (Bendig et al., 2015; Chen et al., 2016; Wang et al., 2017; Maimaitijiang et al., 2019; Li et al., 2020). For instance, spectral indices and plant height were used to predict barley, wheat, and potato AGB (Bendig et al., 2015; Yue et al., 2017; Li et al., 2020); and spectral and structural data fusion was applied for AGB estimation in maize (Wang et al., 2017). In this study, we compared two methods, LASSO regression and PLSR, combining 19 features (Supplementary Table 1) extracted from RGB and multispectral imagery captured with UAV to predict soybean AGB. Our results showed that both methods presented similar performances in all environments (Figure 2). When assessing the importance of the individual variables from the LASSO regression (Supplementary Figure 2), we observed that this method used information from different predictor variables for each environment. For example, the relative importance of CC was higher for 2018_ACRE and 2018_Romney than 2017_ACRE. On the other hand, NDVI was only included in the model to predict AGB at 2017-ACRE. This is also supported by the results of the CV leaving one environment out which indicates that new environments could not be predicted accurately (Supplementary Figure 3). These results provided a solid basis for constructing different models for each environment to enhance the strengths of each imagery feature by the environment.

### Genetic Architecture of Soybean Temporal Above-Ground Biomass

The identification of the genetic causes underlying phenotypic variation is a major step toward crop improvement. By implementing an HTPP that is capable of collecting non-destructive data in large populations throughout the season under actual field conditions, researchers and plant breeders are able to quantify and understand more thoroughly the dynamics of temporal variation of traits and thereby better optimize genotypes through selection in breeding programs (Pauli et al., 2016). It is important to note that the effort and investment in HTTP demand equal effort to properly analyze the data. Nevertheless, the improvement of statistical methodologies to analyze image-based longitudinal phenotypes has not kept pace with the ability to generate high-throughput phenotypic data (Momen et al., 2019). Most of the studies using longitudinal traits mainly performed statistical genetic or genomic analysis for each time point independently (Würschum et al., 2014; Pauli et al., 2016; Xavier et al., 2017; Zhang et al., 2017; Wang et al., 2019; Knoch et al., 2020), ignoring the existing temporal genetic correlation and dependency during trait development. RRM are deemed the most effective alternative to genetically evaluate longitudinal traits in numerous livestock breeding programs (Oliveira et al., 2019a). This approach uses the covariance between each time point with no assumptions of constant variances or correlations, resulting in more accurate breeding values compared to other methods (Sun et al., 2017; Oliveira et al., 2019a). We combined HTP data, high-density genomic information, and RRM to carry out longitudinal analysis and understand the genetics of the development of AGB in soybean. In this context, this study provides the first application of RRM for genomic analyses of longitudinal traits in soybean, as well as the first genetic study on soybean AGB.

Among the RRM tested here, the model using quadratic B-spline with one knot and homogeneous residual variance failed to converge, which indicates that this model did not fit the data well (Supplementary Table 2). The models using fifth-order Legendre polynomial and quadratic B-spline with two knots also did not achieve convergence when heterogeneous residual variance was used, probably because of the higher complexity of the models (i.e., they are more parameterized) and the dataset size. Usually, more parametrized models require a higher number of observations to accurately estimate their parameters (Thoni et al., 1990). As the number of parameters increases, problems with convergence and estimation, as well as an increase in computational demand, can be expected. The model that seemed to be the most suitable to fit the data was the model fitting linear B-spline with two knots and heterogeneous residual variance. Hence, this model was selected to describe the genetic architecture of AGB over time in subsequent analyses.

We observed that the heritability for AGB fluctuates over DAP (Figure 3), indicating that the proportion of genetic variance responsible for the phenotypic variation changes across DAP, which is expected due to differential growth patterns and fluctuation of some environmental variables across development and across three locations. Using RRM on phenotypes collected in a controlled-environment, Campbell et al. (2018) found heritabilities ranging from 0.60 to 0.77 for shoot biomass in rice. Studies using independent analyses of individual time points of phenotypes from controlled-environments found high broad-sense heritabilities for AGB in barley (Neumann et al., 2017), maize (Muraya et al., 2017), and canola (Knoch et al., 2020). Lack of environmental variation throughout growth likely contributes to the high heritabilities observed in these studies. Under the field conditions of multi-environment trials, as in our study, the genetic contribution to the observed phenotypes is both variable and reduced due to environmental fluctuations. Regarding genetic correlation of phenotypes across days (Figure 4), Campbell et al. (2018) and Baba et al. (2020) observed the same trend that we did, where the highest correlations were observed between adjacent time points.

Using RRM allowed us to specify the residual variance structure over time, and what we chose to apply likely contributes to the heritability fluctuations we observed. We grouped interpolated AGB phenotypes with observed phenotypes for the DAP nearest in time, which may not reflect the true residual variance of the longitudinal data. Nonetheless, all models with the heterogeneity of residual variance structure outperformed the models with homogeneous residual variance (Supplementary Table 2), agreeing with other studies (Brito et al., 2017; Campbell et al., 2018). The residual variance is affected by many factors that change with DAP, for instance, as the plants grow the scale of AGB phenotypes increases dramatically from approximately 10 to 940 g/m^2^. Thus, when considering the genetic architecture of longitudinal traits it is crucial to assess the need of a heterogeneous residual variances structure over time points, since there can be improvements in the partition of the total variation, yielding better estimates of genetic parameters (Brito et al., 2017). In this context, it is important to emphasize that this approach is often performed in studies using RRM (Brito et al., 2017; Campbell et al., 2018).

In this study, time was introduced as an additional dimension to association studies enabling the observation of the effects of individual markers over 57 days of soybean AGB development from late vegetative up to mid reproductive stages between 27 and 83 DAP. For longitudinal traits, such as AGB, genetic effects are expected to vary over time and studies have shown that the additive polygenic effects of longitudinal traits are not constant over time (Brito et al., 2017; Oliveira et al., 2019a). The RRM approach improves statistical power to detect loci associated with longitudinal traits over other methods because the entire collection of phenotypic observations is considered, capturing the genetic changes throughout the time period considered (Ning et al., 2017; Oliveira et al., 2019a). Therefore, RRM longitudinal GWAS can detect time-dependent significant SNPs that might not be detected when using independent analyses of individual time points.

We observed SNP effects were generally small and time-specific (Figure 6), and no SNPs had a significant association with soybean AGB throughout the observed time period, suggesting the trait is regulated by small effect loci and their interactions. This highlights the importance of the temporal assessment of longitudinal traits, as many associations could not have been discovered if AGB had been evaluated at the end of the experiment or at individual time points. Previous studies have explored the dynamic genetic architecture of AGB in other crops (Campbell et al., 2017, 2019; Muraya et al., 2017; Knoch et al., 2020), but none at field scale or with high temporal resolution. Campbell et al. (2017) used power function parameters as the pseudo-phenotypes in a multiple-trait GWAS to study AGB in rice during early and active tillering stages. Using RRM, several loci with both transient and persistent effects were found controlling rice AGB during early vegetative development in a green-house (Campbell et al., 2019). Knoch et al. (2020) used time point data and relative growth rates for a GWAS of canola AGB under controlled-environment conditions and observed that several medium and many small effect loci controlled the trait, most of which act during short periods.

Among the selected SNPs positioned within candidate genes in soybean (Table 1), some may have a direct impact on AGB. The *Glyma.02 g064600* candidate gene potentially codes a protein belonging to the Agenet domain family, which is known as chromatin remodeling proteins (Brasil et al., 2015). In *Arabidopsis thaliana*, Agenet/Tudor domain family proteins were associate with regulating gene expression by DNA methylation (Brasil et al., 2015; Zhang et al., 2018). Interestingly, an Agenet domain-containing protein in *A. thaliana* was highly expressed in reproductive tissues and its downregulation delayed flower development timing (Brasil et al., 2015). In our study, the effect of the SNP associates with *Glyma.02 g064600* started to be present at 43 DAP, which overlaps with the average beginning of the blooming (R1) period, and the magnitude of its effects increases with time. Also, on chromosome two, *Glyma.02 g064500* possibly corresponds to rhomboid protein-related that in *A. thaliana* is a putative cellular component in the Golgi apparatus with unknown function. Ban et al. (2019) reported that *Glyma.07 g067900*, which codes a disease resistance protein, was upregulated when studying the regulation of genes in mutant dwarf soybeans related to plant growth. It is known that the over-expression of disease resistance and other immune-responsive genes tend to divert resources to generate protection metabolites, thus reducing overall growth (Ban et al., 2019). *Glyma.07 g071800* is predicted to have biological functions involved in the riboflavin biosynthetic process. In plants, Riboflavin is known to be involved in disease defense (Nie and Xu, 2016), therefore *Glyma.07 g071800* may be associated with the trade-off between the defense response and plant growth as mentioned before. *Glyma.16 g046000* is a putative DEAD/DEAH box helicase. Some proteins of this family are known to play a role in plant growth and development, and in response to stresses in plants (Wang et al., 2000; Zhu et al., 2015). These results improve our understanding of the genetic control of soybean AGB and bridge gaps in understanding the relationship between genotype and phenotype. Further studies are necessary to validate the potential candidate genes and understand their contribution to soybean AGB.

### Potential of Genomic Selection to Improve Soybean Temporal Above-Ground Biomass

Genomic selection has been proved to be a powerful tool in plant and livestock breeding (Meuwissen et al., 2016; Crossa et al., 2017). HTPP allow crop scientists to generate high-quality phenotypic data and effectively characterize large training populations throughout the growing season. Thus, the combination of GS and HTPP has the potential to increase accuracy and throughput, while reducing costs and minimizing labor (Araus et al., 2018). Several studies in animals have demonstrated that RRM improve genomic prediction accuracy of longitudinal traits compared to single-time point and MTM (Oliveira et al., 2019a; Moreira et al., 2020) and more recently, this has been demonstrated in plants (Campbell et al., 2018; Momen et al., 2019).

We evaluated the effectiveness of RRM-based genomic selection for longitudinal soybean AGB. Using CV, we found that it was possible to model longitudinal AGB with RRM (Figure 5). Prediction accuracy varied across DAP, with a decreasing trend over time. Accuracy of GS is dependent on many factors, such as the level of linkage disequilibrium (LD) in the population, effective population size, the number of markers, trait heritability, and the number of QTL influencing the trait (Lin et al., 2014; Wang et al., 2018). Since the LD, population size and number of markers were held constant in our study, the difference in prediction accuracy across DAP can be largely attributed to the differences in heritability. Considering the heritability values, in general, we obtained better prediction accuracy than Campbell et al *(*2018) observed when predicting AGB in rice using RRM. Prediction bias for the GEBVs also varied over DAP, suggesting that selection based on different days produces different results (Supplementary Figure 6). This is in agreement with our GWAS results because it implies that different genes can be expressed by DAP and that selection based on different days can have distinct genetic implications on AGB (Oliveira et al., 2019b). One possible reason for the decrease in prediction accuracy and bias over time could be decreasing quality of the phenotypes as the season progresses and the plot canopy closes, because it is difficult to quantify accurate phenotypic differences between plots. Phenotyping accuracy can be improved by enhancing imagery resolution and adding volume and height metrics. Another reason may be our limited population size (*n* = 383). Increasing population and training set size generally increase the accuracy of predictions, especially for low heritability traits (Goddard, 2009; Wang et al., 2018). Xavier et al. (2016) found that training population size was the most relevant factor in improving prediction accuracy in the SoyNAM population, with optimal populations size between 1,000 and 2000 individuals.

In summary, based on the prediction accuracy and bias, our results indicate that AGB is a potential candidate for genomic selection in soybeans. The ability to predict temporal-based GEBV allows targeting specific intervals in the growing season or selecting plants with specific growth patterns. For instance, increased temperatures and water stress can reduce AGB significantly, resulting in reduction in soybean yield (Jumrani and Bhatia, 2018); using genomic selection to increase AGB during vegetative stages and making the plant more robust may improve stress resilience. Moreover, even if a longitudinal trait itself is not the target of selection, but its genetically correlated to economic traits, such as yield, it has the potential of being used for early indirect selection or to improve genomic prediction accuracy in a MTM (Sun et al., 2017; Moreira et al., 2020). The genetic correlation between longitudinal soybean AGB and grain yield is currently being investigated. Given HTPP’s power to simultaneously collect multiple temporal traits, multiple-trait RRM may be powerful tools for joint genomic prediction of multiple longitudinal traits (Oliveira et al., 2016; Baba et al., 2020; Moreira et al., 2020). Therefore, RRM and HTPP have a great potential to accelerate the rate of genetic gain in soybean breeding programs.

## Data Availability Statement

The raw data supporting the conclusions of this article will be made available by the authors upon request.

## Author Contributions

FM developed the experiment, collected the field data, conducted the statistical analyses, and wrote the manuscript. FM and KR conceived and designed the study. ML assisted with field data collection. KC and BA conducted the multispectral images analyses. GG assisted with AGB phenotypic prediction. HO and LB assisted with the random regression model analyses. HO, LB, and KR critically revised and improved the manuscript. All authors read and approved the manuscript.

## Conflict of Interest

The authors declare that the research was conducted in the absence of any commercial or financial relationships that could be construed as a potential conflict of interest.

## Publisher’s Note

All claims expressed in this article are solely those of the authors and do not necessarily represent those of their affiliated organizations, or those of the publisher, the editors and the reviewers. Any product that may be evaluated in this article, or claim that may be made by its manufacturer, is not guaranteed or endorsed by the publisher.
