# Supplementary material for: High-Throughput Phenotyping and Random Regression Models Reveal Temporal Genetic Control of Soybean Biomass Production
Source: Front Plant Sci. 2021 Sep 3;12:715983. doi: 10.3389/fpls.2021.715983 (PMC8446606; doi:10.3389/fpls.2021.715983)
Supplement: Supplementary file 1 [file Data_Sheet_1.docx]

Supplementary Material

# Supplementary Figures


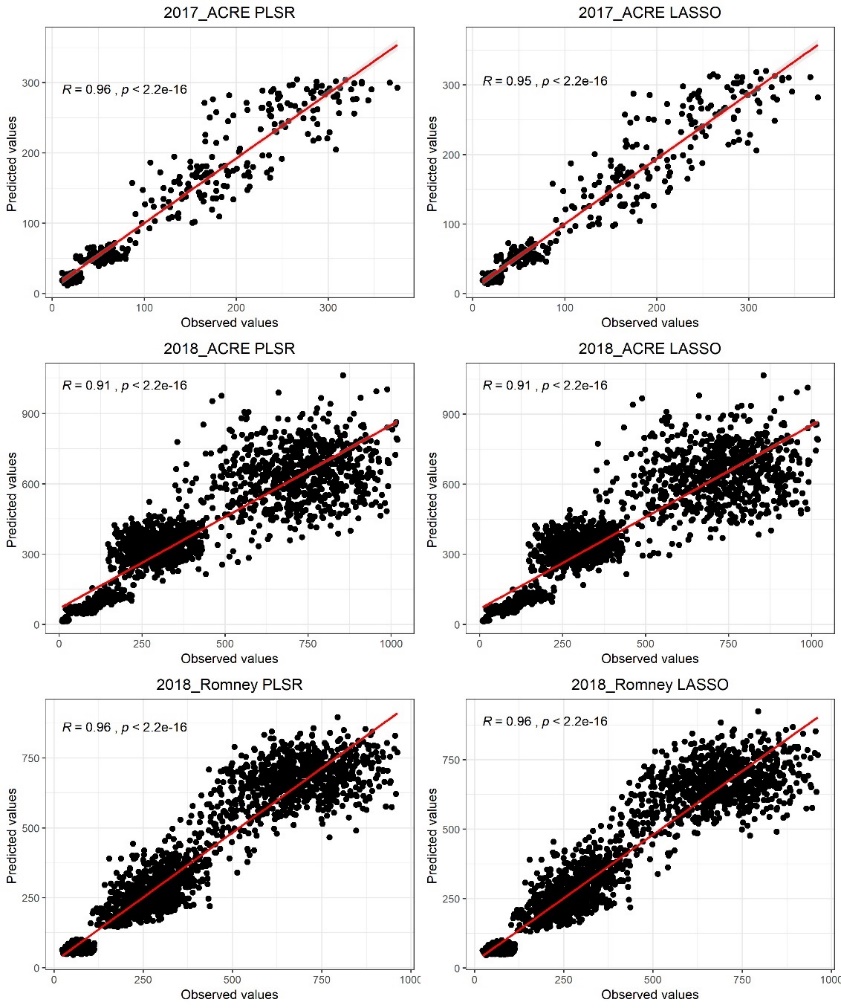


**Supplementary Figure 1.** Correlation (R) plots between observed and predicted above-ground biomass (g/m2). The least absolute shrinkage and selection operator (LASSO) regression and partial least squares regression (PLSR) correlation by environment.


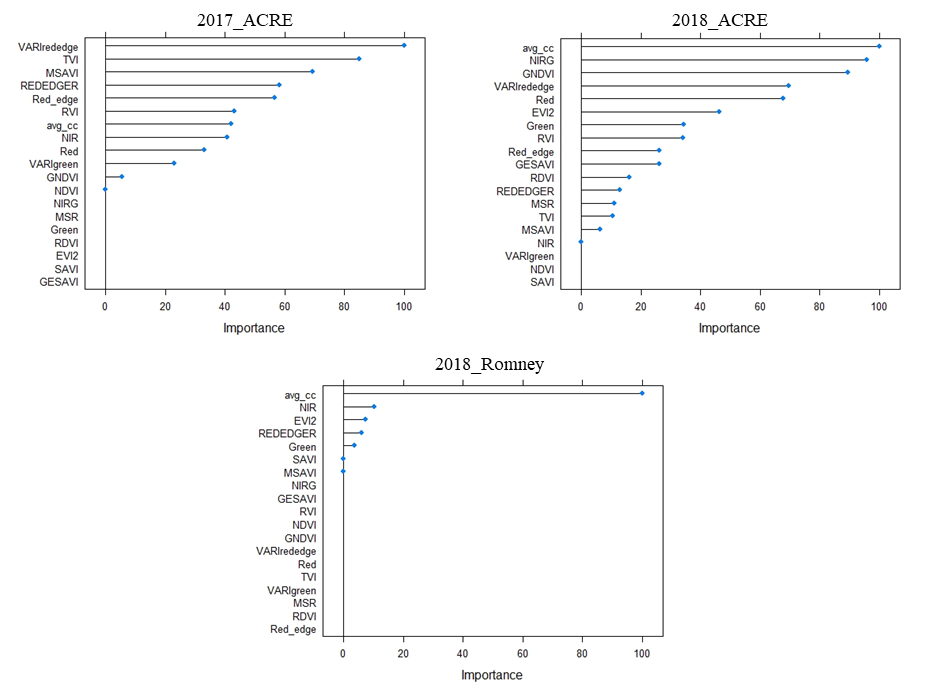


**Supplementary Figure 2.** Variable importance for each environment for the least absolute shrinkage and selection operator (LASSO) regression.


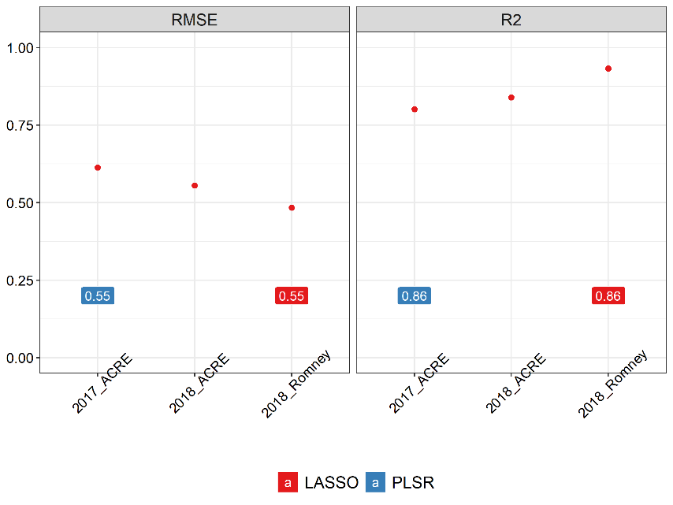


**Supplementary Figure 3.** Performance of above-ground biomass prediction leaving one environment out. Predictions were performed using the least absolute shrinkage and selection operator (LASSO) regression, and the partial least squares regression (PLSR) methods. The performance of predictions was evaluated using the root mean squared error (RMSE) and coefficient of determination (R2).


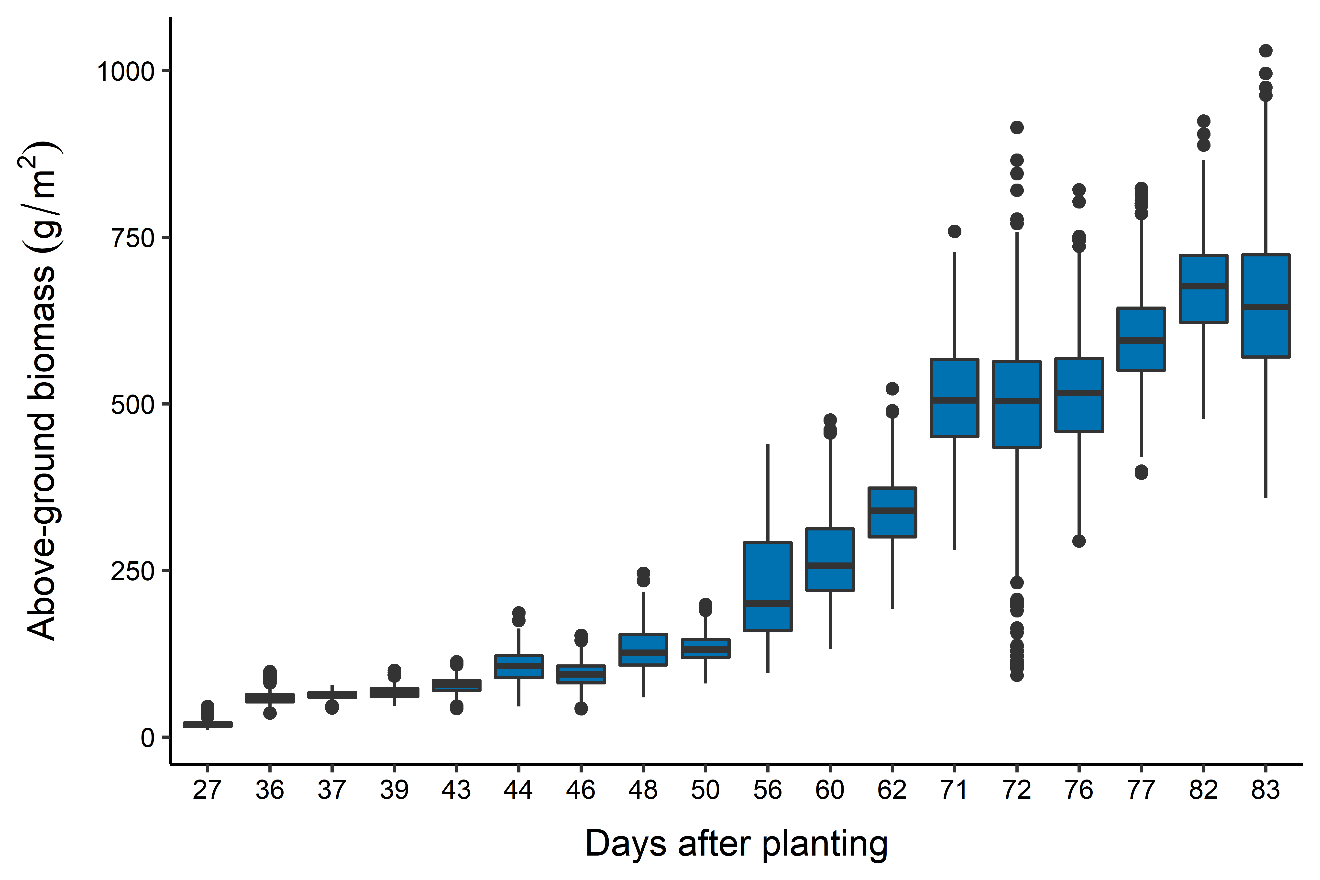


**Supplementary Figure 4.** Predicted above-ground biomass across environments. Phenotypic distributions of predicted above-ground biomass (g/m2) across environments by days after planting using the Least Absolute Shrinkage and Selection Operator (LASSO) Regression. Horizontal lines in the box indicate the median.


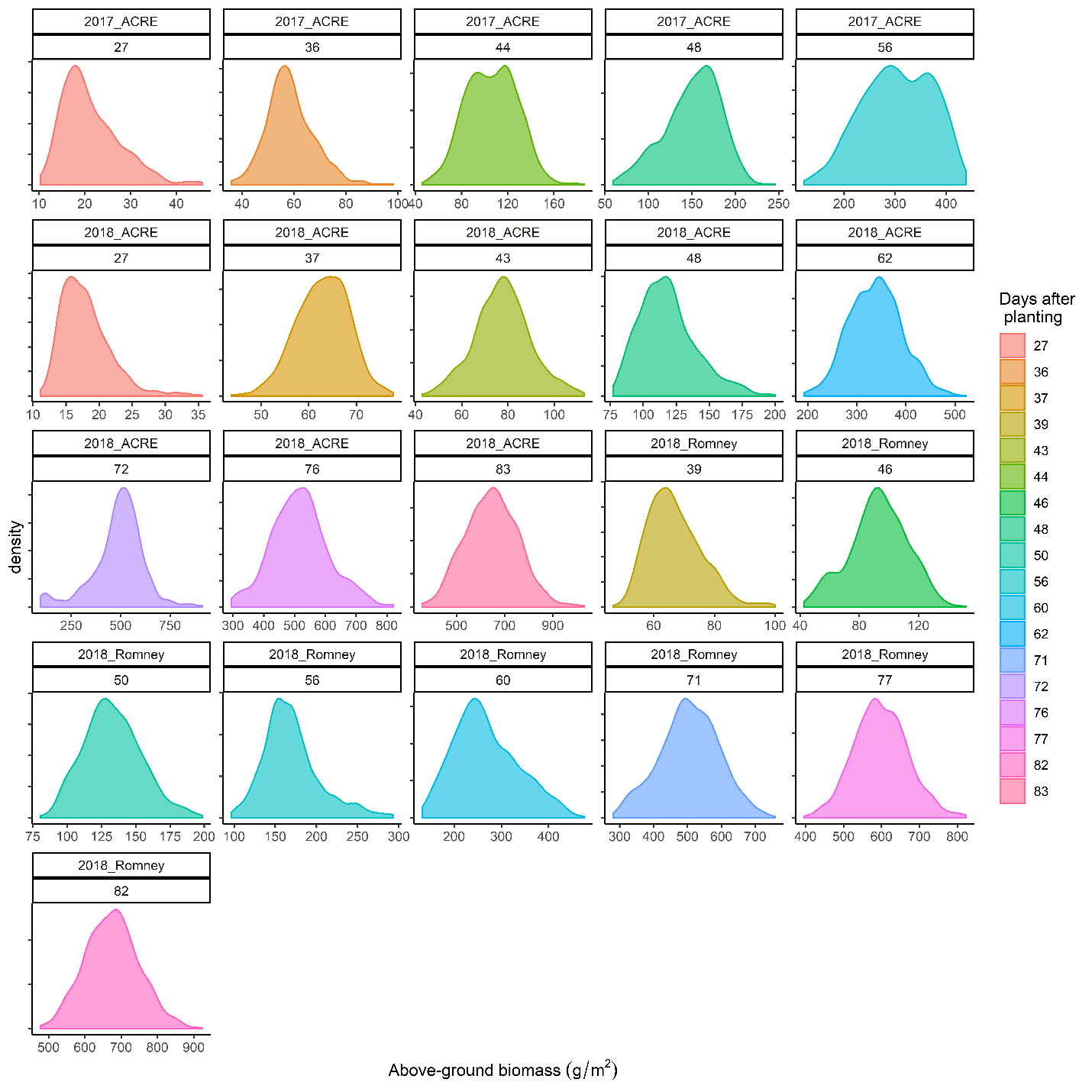


**Supplementary Figure 5.** Predicted above-ground biomass by environment. Phenotypic distribution of predicted above-ground biomass (g/m2) using the Least Absolute Shrinkage and Selection Operator (LASSO) Regression by days after planting for each environment.


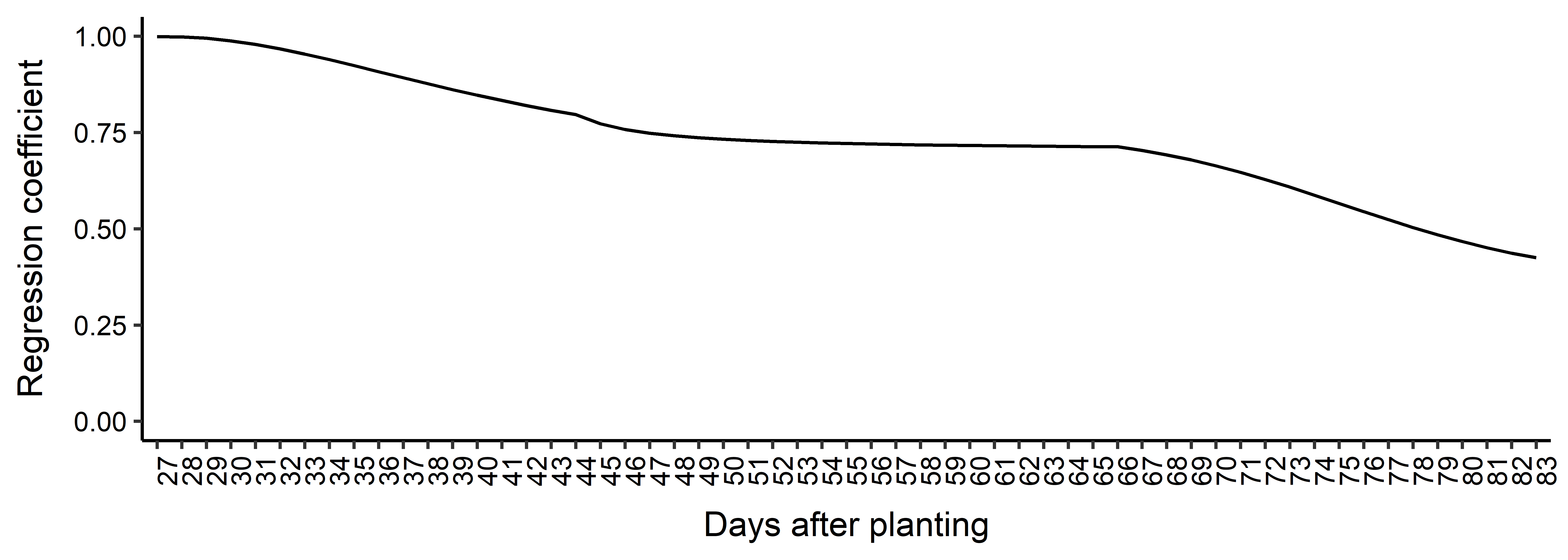


**Supplementary Figure 6.** Selection bias over days after planting. Regression coefficients’ patterns of genomic estimated breeding values for each day after planting.

# Supplementary Tables

**Supplementary Table 1.** Descriptions and formulas of imagery features investigated in this study.

| **Acronym** | **Feature** | **Definition** | **Sensor** | **Citation** |
| --- | --- | --- | --- | --- |
| R_red_ | Reflectance of Red band | R_660_ | MSP | -- |
| R_green_ | Reflectance of Green band | R_550_ | MSP | -- |
| R_rededge_ | Reflectance of Red Edge band | R_735_ | MSP | -- |
| R_NIR_ | Reflectance of Near Infra-Red band | R_790_ | MSP | -- |
| NDVI | Normalized Difference Vegetation Index | (R_NIR_ − R_red_)/ (R_NIR_ + R_red_) | MSP | (Rouse Jr. *et al.* 1974) |
| SAVI | Soil-Adjusted Vegetation Index | (R_NIR_ − R_red_)/ (R_NIR_ + R_red_ + 0.5) * 1.5 | MSP | (Huete 1988) |
| MSAVI | Modified Soil-adjusted Vegetation Index | (2* R_NIR_ + 1-(((2*R_NIR_ +1) ^2)-8*(R_NIR_ - R_red_)) ^0.5)/2 | MSP | (Qi *et al.* 1994) |
| GESAVI | Generalized Soil-adjusted Vegetation Index | ((R_NIR_-1.18) * (R_red_-0.012))/(R_red_+0.35) | MSP | (Gilabert *et al.* 1998) |
| GNDVI | Green-NDVI | (R_NIR_ − R_green_)/ (R_NIR_ + R_green_) | MSP | (Gitelson *et al.* 1996) |
| RVI | Ratio Vegetation Index | (R_NIR_ / R_red_) | MSP | (Jordan 1969) |
| MSR | Modified Simple Ratio Index | ((R_NIR_ − R_red_) - 1)/ √ (R_NIR_ / R_red_ +1) | MSP | (Chen 1996) |
| RDVI | Re-normalized Difference Vegetation Index | (R_NIR_ − R_red_) / √ (R_NIR_ + R_red_) | MSP | (Roujean and Breon 1995) |
| TVI | Transformational Vegetation Index | √ (NDVI + 0.5) | MSP | (Deering *et al.* 1975) |
| GRVI | Green Ratio Vegetation Index | (R_NIR_ / R_green_) | MSP | (Inada 1985) |
| EVI2 | Enhanced Vegetation Index 2 | 2.5*((R_NIR_ - R_red_)/(R_NIR_+2.4*R_red_+1)) | MSP | (Jiang *et al.* 2008) |
| VARI_green_ | Modified Visible Atmospherically Resistant Index- green | (R_green_ − R_red_)/ (R_green_ + R_red_) | MSP | (Gitelson *et al.* 2002) |
| VARI_rededge_ | Modified Visible Atmospherically Resistant Index- red edge | (R_rededge_ − R_red_)/ (R_rededge_ + R_red_) | MSP | (Gitelson *et al.* 2002) |
| REDEDGER | Red edge/green ratio | (R_rededge_ / R_green_) | MSP | -- |
| CC | Canopy Coverage | Percentage of Green pixels/Total pixels | RGB | (Hearst 2019) |

RGB: Red, green, blue. MSP: multispectral. R_i_: reflectance at band i (nanometer).

**Supplementary Table 2.** Random regression models comparison. Akaike’s information criterion (AIC) values calculated for the random regression models tested using homogenous and heterogeneous residual variances.

| **Model ^a^** | **AIC** | |
| --- | --- | --- |
|  | **Homogeneous residual variance** | **Heterogeneous residual variance** |
| Third-order Legendre Polynomial | 165,795.94 | 149,108.17 |
| Fourth-order Legendre Polynomial | 164,767.63 | 148,437.23 |
| Fifth-order Legendre Polynomial | 163,865.17 | - |
| Linear B-spline 1 knot | 165,461.99 | 148,468.87 |
| Linear B-spline 2 knots | 164,922.42 | 147,386.23 |
| Quadratic B-spline 1 knot | - | 148,803.59 |
| Quadratic B-spline 2 knots | 163,851.66 | - |

^a^ Random regression models with respective Legendre Polynomial or B-spline for the fixed curve and for the additive genetic effect. - Model did not achieve the convergence criterium of $\mathbf{10}^{\mathbf{-12}}$.

**Supplementary Table 3.** Candidate genes. List of candidate genes for all selected SNP associated with above-ground biomass in soybean according to SoyBase (SoyBase.org).

| **Duration Category** | **SNP** | **Chr.** | **Pos. (bp)** | **Selected candidate genes** | **Database ID** | **Annotation Description** |
| --- | --- | --- | --- | --- | --- | --- |
| Long | 2:5777782 | 2 | 5,777,782 | *Glyma.02g064300* | AT2G02990.1 | ribonuclease 1 |
|  |  |  |  | *Glyma.02g064400* | AT2G02990.1 | ribonuclease 1 |
|  |  |  |  | *Glyma.02g064500* | AT3G07950.1 | rhomboid protein-related |
|  |  |  |  | *Glyma.02g064600* | AT1G09320.1 | agenet domain-containing protein |
|  |  |  |  | *Glyma.02g064700* | AT3G54890.1 | photosystem I light harvesting complex gene 1 |
|  |  |  |  | *Glyma.02g064800* | AT3G26935.1 | DHHC-type zinc finger family protein |
|  |  |  |  | *Glyma.02g064900* | AT1G48160.1 | signal recognition particle 19 kDa protein, putative / SRP19, putative |
| Short | 3:5150181 | 3 | 5,150,181 | *Glyma.03g040800* | AT3G23270.1 | regulator of chromosome condensation (RCC1) family with FYVE zinc finger domain |
|  |  |  |  | *Glyma.03g040900* | AT5G01750.2 | protein of unknown function (DUF567) |
| Long | 3:10014176 | 3 | 10,014,176 | *Glyma.03g063400* | AT3G60470.1 | plant protein of unknown function (DUF247) |
| Intermittent | 3:14985662 | 3 | 14,985,662 | NA | - | - |
| Short | 4:10352467 | 4 | 10,352,467 | NA | - | - |
| Mid | 4:13022455 | 4 | 13,022,455 | *Glyma.04g114400* | GO:0016021 | integral component of membrane |
|  |  |  |  | *Glyma.04g114500* | - | - |
|  |  |  |  | *Glyma.04g114600* | - | - |
|  |  |  |  | *Glyma.04g114700* | - | - |
| Short | 4:14549891 | 4 | 14,549,891 | NA | - | - |
| Short | 5:3808314 | 5 | 3,808,314 | *Glyma.05g042400* | AT5G50210.1 | quinolinate synthase |
|  |  |  |  | *Glyma.05g042500* | AT4G13310.1 | cytochrome P450, family 71, subfamily A, polypeptide 20 |
|  |  |  |  | *Glyma.05g042600* | AT4G13310.1 | cytochrome P450, family 71, subfamily A, polypeptide 20 |
|  |  |  |  | *Glyma.05g042700* | AT4G24740.1 | FUS3-complementing gene 2 |
| Long | 7:6108702 | 7 | 6,108,702 | *Glyma.07g067500* | AT5G41460.1 | protein of unknown function (DUF604) |
|  |  |  |  | *Glyma.07g067600* | AT3G30280.1 | HXXXD-type acyl-transferase family protein |
|  |  |  |  | *Glyma.07g067700* | AT1G01580.1 | ferric reduction oxidase 2 |
|  |  |  |  | *Glyma.07g067800* | AT4G12010.1 | disease resistance protein (TIR-NBS-LRR class) family |
|  |  |  |  | *Glyma.07g067900* | AT5G17680.1 | disease resistance protein (TIR-NBS-LRR class), putative |
|  |  |  |  | *Glyma.07g068000* | AT3G61460.1 | brassinosteroid-responsive RING-H2 |
| Mid | 7:6523718 | 7 | 6,523,718 | *Glyma.07g071500* | AT3G61350.1 | SKP1 interacting partner 4 |
|  |  |  |  | *Glyma.07g071600* | AT3G61350.1 | SKP1 interacting partner 4 |
|  |  |  |  | *Glyma.07g071700* | - | - |
|  |  |  |  | *Glyma.07g071800* | AT4G20960.1 | cytidine/deoxycytidylate deaminase family protein |
|  |  |  |  | *Glyma.07g071900* | AT3G61320.1 | bestrophin-like protein |
|  |  |  |  | *Glyma.07g072100* | AT4G00710.1 | BR-signaling kinase 3 |
| Long | 7:13739807 | 7 | 13,739,807 | *Glyma.07g119600* | PF01107 | viral movement protein (MP) |
|  |  |  |  | *Glyma.07g119700* | AT5G59540.1 | 2-oxoglutarate (2OG) and Fe(II)-dependent oxygenase superfamily protein |
|  |  |  |  | *Glyma.07g119800* | AT1G02180.1 | ferredoxin-related |
|  |  |  |  | *Glyma.07g119900* | AT1G22860.1 | vacuolar sorting protein 39 |
|  |  |  |  | *Glyma.07g120000* | AT3G31430.1 |  |
| Short | 7:15340513 | 7 | 15,340,513 | *Glyma.07g128000* | AT4G26200.1 | 1-amino-cyclopropane-1-carboxylate synthase 7 |
|  |  |  |  | *Glyma.07g128100* | AT4G29510.1 | arginine methyltransferase 11 |
|  |  |  |  | *Glyma.07g128200* | AT4G29520.1 | - |
|  |  |  |  | *Glyma.07g128300* | AT5G11780.1 | - |
|  |  |  |  | *Glyma.07g128400* | AT5G56750.1 | N-MYC downregulated-like 1 |
|  |  |  |  | *Glyma.07g128500* | AT4G26180.1 | mitochondrial substrate carrier family protein |
|  |  |  |  | *Glyma.07g128600* | AT2G15690.1 | tetratricopeptide repeat (TPR)-like superfamily protein |
|  |  |  |  | *Glyma.07g128700* | AT5G56780.1 | effector of transcription2 |
| Long | 7:27576963 | 7 | 27,576,963 | NA | - | - |
| Mid | 8:21488582 | 8 | 21,488,582 | *Glyma.08g247700* | AT3G29770.1 | methyl esterase 11 |
|  |  |  |  | *Glyma.08g247600* | AT4G26190.1 | haloacid dehalogenase-like hydrolase (HAD) superfamily protein |
| Mid | 8:21970879 | 8 | 21,970,879 | *Glyma.08g250700* | AT3G02310.1 | K-box region and MADS-box transcription factor family protein |
|  |  |  |  | *Glyma.08g250800* | AT5G60910.1 | AGAMOUS-like 8 |
|  |  |  |  | *Glyma.08g250900* | AT3G02300.1 | regulator of chromosome condensation (RCC1) family protein |
| Short | 11:5076944 | 11 | 5,076,944 | *Glyma.11g067500* | AT5G66750.1 | chromatin remodeling 1 |
|  |  |  |  | *Glyma.11g067700* | AT3G50660.1 | cytochrome P450 superfamily protein |
|  |  |  |  | *Glyma.11g067800* | AT5G66760.1 | succinate dehydrogenase 1-1 |
| Mid | 11:6004142 | 11 | 6,004,142 | *Glyma.11g079500* | AT4G35920.1 | PLAC8 family protein |
|  |  |  |  | *Glyma.11g079600* | AT1G75950.1 | S phase kinase-associated protein 1 |
|  |  |  |  | *Glyma.11g079700* | AT3G51320.1 | pentatricopeptide repeat (PPR) superfamily protein |
|  |  |  |  | *Glyma.11g079800* | PF03966 | Trm112p-like protein |
|  |  |  |  | *Glyma.11g079900* | AT3G51325.1 | RING/U-box superfamily protein |
|  |  |  |  | *Glyma.11g080000* | AT4G35900.1 | basic-leucine zipper (bZIP) transcription factor family protein |
|  |  |  |  | *Glyma.11g080100* | AT4G35890.1 | winged-helix DNA-binding transcription factor family protein |
| Mid | 12:3072635 | 12 | 3,072,635 | *Glyma.12g042300* | AT2G17080.1 | *Arabidopsis* protein of unknown function (DUF241) |
|  |  |  |  | *Glyma.12g042400* | AT4G39210.1 | glucose-1-phosphate adenylyltransferase family protein |
|  |  |  |  | *Glyma.12g042500* | AT2G16365.1 | F-box family protein |
|  |  |  |  | *Glyma.12g042600* | AT4G25730.1 | FtsJ-like methyltransferase family protein |
|  |  |  |  | *Glyma.12g042700* | AT4G34540.1 | NmrA-like negative transcriptional regulator family protein |
|  |  |  |  | *Glyma.12g042800* | AT2G18300.2 | basic helix-loop-helix (bHLH) DNA-binding superfamily protein |
| Intermittent | 13:2112345 | 13 | 2,112,345 | *Glyma.13g007700* | AT3G51690.1 | PIF1 helicase |
|  |  |  |  | *Glyma.13g007800* | AT1G02960.2 | - |
|  |  |  |  | *Glyma.13g007900* | AT4G12680.1 | integral component of membrane |
| Long | 13:24980935 | 13 | 24,980,935 | *Glyma.13g137000* | AT3G09670.1 | tudor/PWWP/MBT superfamily protein |
|  |  |  |  | *Glyma.13g137100* | AT5G02960.1 | ribosomal protein S12/S23 family protein |
|  |  |  |  | *Glyma.13g137200* | AT2G37080.1 | ROP interactive partner 3 |
| Short | 13:37339900 | 13 | 37,339,900 | *Glyma.13g271100* | AT3G14980.1 | acyl-CoA N-acyltransferase with RING/FYVE/PHD-type zinc finger protein |
|  |  |  |  | *Glyma.13g271200* | AT1G53280.1 | class I glutamine amidotransferase-like superfamily protein |
|  |  |  |  | *Glyma.13g271300* | AT1G53270.1 | ABC-2 type transporter family protein |
|  |  |  |  | *Glyma.13g271400* | AT3G15000.1 | cobalt ion binding |
|  |  |  |  | *Glyma.13g271500* | AT5G20040.1 | isopentenyltransferase 9 |
| Long | 14:47457673 | 14 | 47,457,673 | *Glyma.14g208600* | AT2G40510.1 | ribosomal protein S26e family protein |
|  |  |  |  | *Glyma.14g208700* | AT5G37150.1 | P-loop containing nucleoside triphosphate hydrolases superfamily protein |
|  |  |  |  | *Glyma.14g208800* | - | - |
|  |  |  |  | *Glyma.14g208900* | AT1G17200.1 | uncharacterised protein family (UPF0497) |
|  |  |  |  | *Glyma.14g209000* | AT5G56550.1 | oxidative stress 3 |
|  |  |  |  | *Glyma.14g209100* | GO:0015979 | photosynthesis |
|  |  |  |  | *Glyma.14g209200* | AT1G31240.1 | bromodomain transcription factor |
| Intermittent | 15:10511120 | 15 | 10,511,120 | *Glyma.15g130600* | AT2G34730.1 | myosin heavy chain-related |
|  |  |  |  | *Glyma.15g130700* | GO:0016020 | membrane |
|  |  |  |  | *Glyma.15g130800* | AT1G30580.1 | GTP binding |
|  |  |  |  | *Glyma.15g130900* | AT4G20940.1 | leucine-rich receptor-like protein kinase family protein |
| Long | 15:13201754 | 15 | 13,201,754 | *Glyma.15g157100* | AT5G44030.1 | cellulose synthase A4 |
|  |  |  |  | *Glyma.15g157200* | AT3G27160.1 | ribosomal protein S21 family protein |
|  |  |  |  | *Glyma.15g157300* | AT4G20040.1 | pectin lyase-like superfamily protein |
|  |  |  |  | *Glyma.15g157400* | AT5G05280.1 | RING/U-box superfamily protein |
|  |  |  |  | *Glyma.15g157500* | AT2G32260.1 | phosphorylcholine cytidylyltransferase |
|  |  |  |  | *Glyma.15g157700* | AT3G17365.1 | S-adenosyl-L-methionine-dependent methyltransferases superfamily protein |
| Short | 15:36306421 | 15 | 36,306,421 | *Glyma.15g217500* | AT1G30820.1 | CTP synthase family protein |
| Mid | 15:36870472 | 15 | 36,870,472 | NA | - | - |
| Long | 16:4353954 | 16 | 4,353,954 | *Glyma.16g045700* | AT3G26680.1 | DNA repair metallo-beta-lactamase family protein |
|  |  |  |  | *Glyma.16g045800* | AT5G13890.1 | family of unknown function (DUF716) |
|  |  |  |  | *Glyma.16g045900* | AT5G46860.1 | syntaxin/t-SNARE family protein |
|  |  |  |  | *Glyma.16g046000* | AT3G02060.2 | DEAD/DEAH box helicase, putative |
|  |  |  |  | *Glyma.16g046200* | AT3G02050.1 | K+ uptake transporter 3 |
| Mid | 16:29044334 | 16 | 29,044,334 | *Glyma.16g133600* | AT3G50930.1 | cytochrome BC1 synthesis |
|  |  |  |  | *Glyma.16g133700* | ATMG01170.1 | ATPase, F0 complex, subunit A protein |
|  |  |  |  | *Glyma.16g133800* | AT1G19630.1 | cytochrome P450, family 722, subfamily A, polypeptide 1 |
| Short | 18:6899324 | 18 | 6,899,324 | *Glyma.18g072800* | AT1G55760.1 | BTB/POZ domain-containing protein |
|  |  |  |  | *Glyma.18g073100* | AT1G55790.1 | domain of unknown function (DUF2431) |
|  |  |  |  | *Glyma.18g073200* | AT4G26490.1 | late embryogenesis abundant (LEA) hydroxyproline-rich glycoprotein family |
|  |  |  |  | *Glyma.18g073300* | AT5G08520.1 | duplicated homeodomain-like superfamily protein |
|  |  |  |  | *Glyma.18g073400* | AT4G26510.1 | uridine kinase-like 4 |
| Intermittent | 18:59148882 | 18 | 59,148,882 | *Glyma.18g302000* | AT2G33510.1 | - |
|  |  |  |  | *Glyma.18g302100* | AT2G39290.1 | phosphatidylglycerolphosphate synthase 1 |
|  |  |  |  | *Glyma.18g302200* | AT4G30860.1 | SET domain group 4 |
|  |  |  |  | *Glyma.18g302300* | AT2G01910.2 | microtubule associated protein (MAP65/ASE1) family protein |

Chr: Chromosome, Pos (bp): position in base pair. NA: no positional candidate gene found within ± 25kb. - Annotation not available.
